# Long-range ordering of two-dimensional wide bandgap tantalum oxide nanosheets in printed films†

**DOI:** 10.1039/d1tc00801c

**Published:** 2021-04-09

**Authors:** Melvin A. Timmerman, Rui Xia, Yang Wang, Kai Sotthewes, Mark Huijben, Johan E. ten Elshof

**Affiliations:** University of Twente, MESA+ Institute for Nanotechnology P.O. Box 217 7500 AE Enschede The Netherlands j.e.tenelshof@utwente.nl

## Abstract

Two-dimensional oxide materials are a well-studied, interesting class of materials, enabled by the fact that their bulk layered metal oxides, such as titanates and niobates, can be easily exfoliated within minutes into 2D nanosheets. However, some promising oxide materials, such tantalum oxide, are much more difficult to delaminate, taking several weeks, due to the higher charge density resulting in stronger Coulombic interactions between the layers. This intrinsic constraint has limited detailed studies for exploiting the promising properties of tantalum oxide 2D nanosheets towards enhanced catalysis and energy storage. Here, we have studied in detail the exfoliation mechanism of high charge density 2D materials, specifically tantalum oxide (TaO_3_) nanosheets. Optimization of tetrabutylphosphonium hydroxide (TBPOH) as the exfoliation agent in a 2 : 1 ratio to HTaO_3_ has resulted in a dramatic reduction of the exfoliation time down to only 36 hours at 80 °C. Furthermore, single monolayers of TaO_3_ nanosheets with >95% coverage have been achieved by Langmuir–Blodgett deposition, while thicker layers (ranging from several tens of nanometers up to microns) exhibiting long-range ordering of the present nanosheets have been realized through inkjet printing. Interestingly, scanning tunneling microscopy analysis indicated a wide bandgap of ∼5 eV for the single TaO_3_ nanosheets. This value is significantly higher than the reported values between 3.5 and 4.3 eV for the layered RbTaO_3_ parent compound, and opens up new opportunities for 2D oxide materials.

## Abstract 2

The exfoliation mechanism of high charge density 2D tantalum oxide nanosheets was optimized. Single monolayers and thicker thin films were realized and the bandgap of single nanosheets was measured electrically.

## Graphical abstract

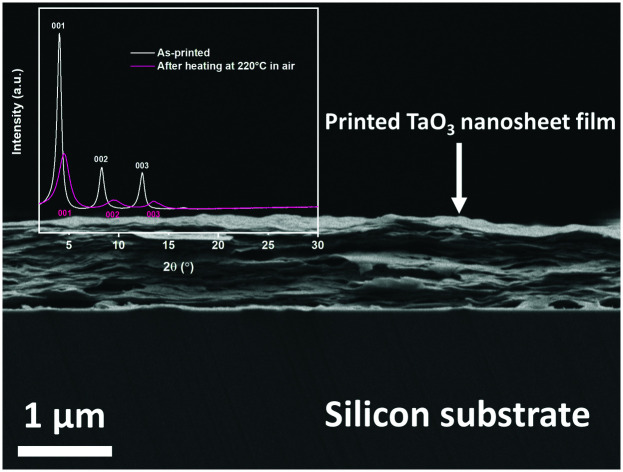

## Introduction

While two-dimensional (2D) materials have been investigated for a long time, the interest in these materials has peaked in the last two decades.^1,2^ The discovery of graphene and its interesting properties boosted the exploration of other 2D materials, often termed nanosheets.^3,4^ Nanosheets have lateral sizes into the micrometer range, but their thicknesses are a couple of orders of magnitude smaller (single nanometer range). This results in a huge area to volume ratio, which is interesting for various areas of application, such as heterogeneous catalysis and energy storage.^5^ Moreover, nanosheets may exhibit interesting physical properties that are different compared to the layered bulk materials from which they were exfoliated. For example, exfoliated Ti_0.87_O_2_ nanosheets, that are as thin as one nanometer, have been proposed as low leakage dielectric films.^6^ Bulk titania has a much lower permittivity than the 2D form. Similarly, TaO_3_ nanosheets have been reported to exhibit a large optical bandgap of ∼5 eV,^7^ whereas bulk Ta_2_O_5_ and the layered RbTaO_3_ phase, from which TaO_3_ nanosheets are made, averages on a bandgap of ∼4 eV.^8–11^ Another study has shown that TaO_3_ nanosheets can contribute to reduce the electrode–electrolyte interfacial resistance in lithium ion batteries.^7^ 2D tantalum oxide is thus an interesting material to explore further.

Various layered materials can be delaminated into nanosheets, *e.g.* clays, hydroxides and metal oxides.^12^ Several layered metal oxides are easily exfoliated, within minutes, such as titanates and niobates.^13^ However, not all metal oxides delaminate readily. For example, tantalum oxide nanosheets have been reported to take roughly three weeks at room temperature to exfoliate from its parent compounds HTaO_3_.^14^ TaO_3_ nanosheets exhibit a relatively high charge density of −1.6 C m^−2^ on their basal plane, while much more easily to exfoliate lepidocrocite-type titanate nanosheets show a charge density of −0.99 C m^−2^,^15^ and RuO_2_ nanosheets have a charge density of −0.46 C m^−2^, calculated on the basis of data from ref. 16 For TaO_3_ this results in stronger Coulombic interactions between the interlayer cations and the TaO_3_^−^ layers. Lower charge densities lead to a weaker electrostatic force between the layers, whereas higher charge densities require more energy to exfoliate a layered intercalation phase.^17–20^ Limited research has been done on the exfoliation of this material, and films consisting of tantalum oxide nanosheets fully covering a substrate have not yet been reported. To realize (patterned) thin films from TaO_3_ nanosheets using inkjet printing, dispersions with a relatively high concentration of nanosheets are needed.

The goal of the research presented here was to better understand the exfoliation mechanism of high surface charge density 2D materials, specifically tantalum oxide TaO_3_^−^. By employing different exfoliating agents, their ratios to the layered material and the exfoliation temperature, the exfoliation procedure was optimized. Fully covering monolayer films of nanosheets were made using Langmuir–Blodgett deposition, as well as layered thin films using inkjet printing. The bandgap of single TaO_3_ nanosheets was electrically determined using scanning tunneling microscopy (STM), to demonstrate the promising functionality for future applications in Li-ion batteries and high-*k* films.

## Experimental

RbTaO_3_ was synthesized following Fukuda *et.al.*^14^ Rb_2_CO_3_ (99.8% Sigma Aldrich) and Ta_2_O_5_ (99% Sigma Aldrich) in a molar ratio of 1.02 : 1 were mixed on a roller bench for 1 day, ground and again mixed for 1 day on a roller bench. In a platinum crucible the mixture was calcined at 1173 K for 20 hours. The resulted powder (RbTaO_3_) was washed 3 times with water and dried in air at room temperature. Protonation of RbTaO_3_ was conducted using 1 M HCl solution (100 cm^3^ g^−1^) for 3 days, where the acid solution was refreshed daily. The resulting HTaO_3_ was used for exfoliation reactions and (LB and inkjet printing) depositions.

The exfoliation process was conducted as follows. 1 g HTaO_3_ was mixed with 100 ml water and 0.61 ml TBPOH (40 wt% in water Sigma Aldrich) (1 : 2 molar ratio of HTaO_3_ to TBPOH) for 36 h in an oil bath at 80 °C. This stock solution was used to make solutions for LB-depositions and inks for inkjet printing.

For LB deposition firstly 14 ml of the stock solution was centrifuged at 1000 rpm for 225 seconds to separate all unreacted residues as sediment. The top 12 ml was added to 40 ml H_2_O to complete the LB solution. The LB trough (KSV Minimicro, a Teflon trough with an active surface area of 100 cm^2^, length 195 × width 51 × depth 4 mm^3^ and a dipping well length 10 × width 28 × depth 28 mm^3^, leading to a volume of 48 cm^3^) was filled with this solution until it was completely filled. The trough with the solution was allowed to stabilize for 5 min. A silicon substrate was vertically lowered in the solution, after which the solution was again stabilized for 5 min. After stabilization the barriers were compressed, thereby increasing the surface pressure. When the surface pressure reached a plateau, the compression was stopped and the substrate was slowly lifted out of the trough. During lift-up, the surface pressure was kept constant by adjusting the barriers. This yielded fully covered substrates with TaO_3_ nanosheets.

Inks for inkjet printing were made using the same stock solution. 16 ml of the stock solution was centrifuged at 1000 rpm for 230 seconds, to separate the unreacted residues. The top 14.4 ml was separated and again centrifuged at 15 000 rpm for 1 h. The liquid top part was poured out and the sedimented bottom part was redispersed in 2.4 ml Triton (0.06 mg ml^−1^ diluted from Triton^TM^ X-100 Sigma Aldrich) and ultrasonicated for one hour. The top redispersed part was poured into a vial and the bottom sediment was discarded. To increase the viscosity of the ink, 10 wt% of propylene glycol was added to the vial. 2 ml of this ink was put in a cartridge and used for inkjet printing with a Dimatix Fujifilm (DMP 2800) printer operated by the software Dimatix Drop Manager Version 2.0.0.1. The printer platen was kept at a temperature of 50 °C, and the drop spacing was 20 μm. The substrates were treated with 0.06 mg ml^−1^ Triton for 2 min prior printing.

X-Ray diffractograms (using a Bruker D2 Phaser) were made of the RbTaO_3_ powder, HTaO_3_ powder and inkjet printed thin films, to confirm the successful synthesis of the precursors and to observe the layered ordering of the inkjet printed films.

Atomic force microscopy (using a Bruker Dimension Icon AFM) was used to make AFM images of the LB-deposited monolayers on silicon substrates to determine the coverage of the substrate and the height of the single nanosheets. The data was analyzed using Gwyddion v2.56 software.

High-resolution scanning electron microscopy (using an HRSEM; Zeiss MERLIN) was conducted on the inkjet printed films to observe the morphology of the layered system.

Scanning tunneling microscopy (STM) was used to determine the band gap of individual nanosheets. The STM measurements are performed in the Nanosurf EasyScan 2 under ambient conditions using PtIr tips. Silicon substrates with a platinum conductive layer on top were covered for 50% with a monolayer of TaO_3_ nanosheets using the LB deposition technique. The platinum conductive layer was put in contact with the current collector using silver paste on the side of the substrate. Several scans were made on single nanosheets and on the bare platinum surface for reference.

## Results and discussion

### Synthesis

**Fig. 1:**
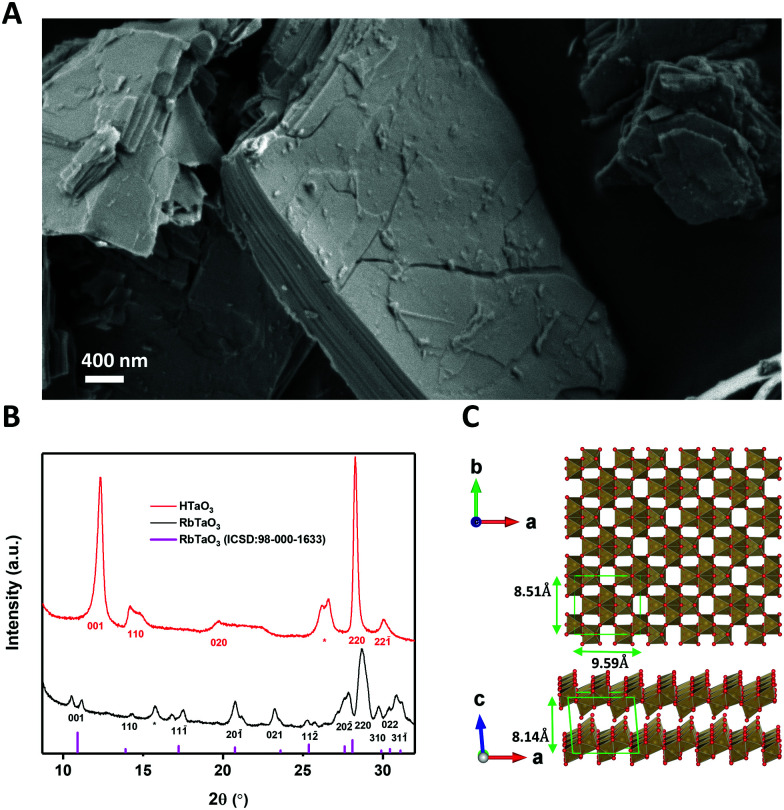
(A) SEM image of the layered crystal structure of HTaO_3_. (B) X-ray diffractograms for RbTaO_3_ and HTaO_3_ and diffraction model of RbTaO_3_. The peaks that could not be identified are indicated with an asterisk. (C) The top- and side-view of the Vesta model of two TaO_3_ nanosheets.

RbTaO_3_ powder was made *via* solid-state synthesis using Ta_2_O_5_ and Rb_2_CO_3_ as reported elsewhere.^14^ In order to be able to exfoliate this material, the interlayer rubidium ions need to be replaced by protons. This weakens the interlayer bond strength and allows the subsequent acid–base reaction to start the exfoliation.^13^ This was done by ion-exchange *via* protonation using HNO_3_, which yields HTaO_3_. A SEM image of the layered structure of the HTaO_3_ powder is shown in Fig. 1(A). Fig. 1(B) shows X-ray diffractograms of RbTaO_3_ and HTaO_3_, Fig. 1(C) shows a top- and side-view of the Vesta model of two TaO_3_ nanosheets.

The XRD peaks of RbTaO_3_ were confirmed using a VESTA model calculation of the expected reflection angles of the parent compound and were also found to be similar to literature data,^14^ confirming the successful synthesis of single-phase RbTaO_3_. All *(hkl)* reflections of RbTaO_3_ where *l* > 0 were split or had a small shoulder, for example the (001) peak at 2*θ* ∼ 11° is split into two peaks. This could be explained by the presence of both intergalleries where rubidium atoms are already fully exchanged by protons (with or without H_2_O solvation shell), and other intergalleries that have remained unaffected. In such a case peaks at different 2*θ* can be present. This phenomenon was also reported in similar studies with layered clays, for example fluorotetrasilicic mica, where (001) peak splitting occurred in diffractograms with different intercalation states.^21^ The as-obtained RbTaO_3_ was then protonated to HTaO_3_. Our diffractogram of HTaO_3_ is similar to XRD data in literature, confirming the successful complete protonation of RbTaO_3_ to yield HTaO_3_.^14^ However, XPS data indicated some Rb atoms still present (Fig. S1, ESI†). XRF analysis was used to quantify the Rb: Ta ratio of 0.044 : 1 (Table S1, ESI†). Please note the absence of peak splitting (as opposed to RbTaO_3_), again most noticeably in the (001) peak in Fig. 1(B). The (001) peak of HTaO_3_ occurs at higher 2*θ* than in RbTaO_3_, and does not have a remnant peak or shoulder at lower 2*θ* coinciding with the (001) peak of RbTaO_3_. This supports the suggestion that the Rb atoms are randomly distributed in the intergallery layers, without causing visible reflections in the XRD data. The (001) reflection shift is indicative of a decrease of the interlaying spacing by 1.2 Å. This is likely due to the replacement of Rb^+^ ions by protons, where protons, including their H_2_O solvation shell, cause the individual layers to come somewhat closer to each other than is possible with Rb^+^ as intercalants.

### Exfoliation of HTaO_3_

The reported exfoliation procedure of HTaO_3_ that takes three weeks to complete was successfully reproduced.^14^ One of the aims of the present study was to reduce the exfoliation time, and to determine the kinetics of the exfoliation process at different temperatures, molar ratios (1 : 1 – 1 : 2) between HTaO_3_ and the exfoliating agents, *i.e.* tetrabutyl ammonium hydroxide (TBAOH) and tetrabutylphosphonium hydroxide (TBPOH). (Tetraethyl and tetramethyl ammonium hydroxide, TEAOH and TMAOH, respectively, were also tried, but they did not yield a high concentration of nanosheets and are therefore not considered further). Instead of monitoring the bulk concentration of nanosheets as a measure of the degree of exfoliation (as is commonly done, *e.g.* by UV-vis analysis), we monitored the surface pressure of a nanosheet dispersion in a Langmuir trough as a measure for the interfacial concentration of nanosheets of that solution. In short, the Langmuir–Blodgett (LB) method uses a Wilhelmy plate to measure the surface pressure of a solution or dispersion. By compressing the outer LB barriers of the trough towards each other, the liquid/air surface area of the trough decreases, thereby concentrating all nanosheets that are floating at the liquid–air interface. Once the interfacial concentration is so high, that the nanosheets interact directly with each other, a notably higher surface pressure is recorded. Saturation of the surface pressure at a high value occurs at a high degree of barrier compression, which indicates that the nanosheets at the liquid–air interface have formed a densely-packed monolayer. When the degree of compression is relatively low when the surface pressure increases sharply, the concentration of nanosheets at this liquid–air interface is relatively high. Linear extrapolation of the pressure *versus* compression curve to zero surface pressure yields the lift-up point (LUP), originally defined by Yuan *et al.* to approximate the nanosheet surface concentration.^13^ Hence, when the LUP is large, this corresponds to a high surface concentration of nanosheets. By systematically changing the exfoliating agent, the molar ratio between exfoliation agent and protonated tantalate, and the exfoliation temperature, the surface concentration of nanosheets can thus be compared, which allows us to compare different exfoliation protocols.

**Fig. 2:**
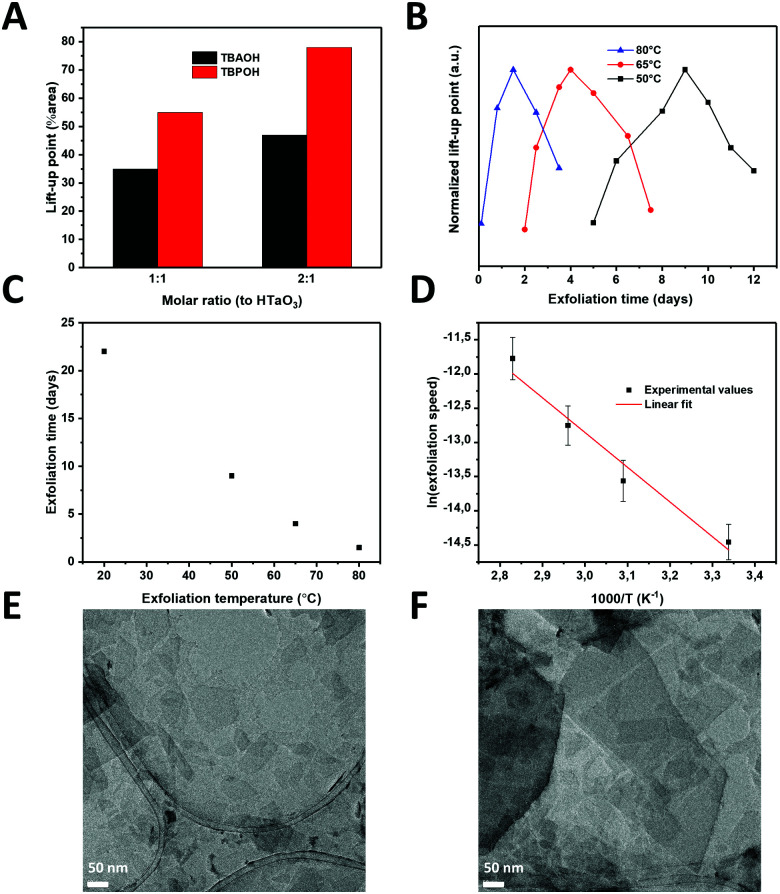
(A) Lift-up point of TaO_3_ nanosheets in solution *versus* the molar ratio of exfoliating agent (TBAOH or TBPOH) to HTaO_3_. (B) Normalized lift-up point of TaO_3_ nanosheets in solution *versus* the exfoliation time for different temperatures. (C) Exfoliation time *versus* the exfoliation temperature. (D) Arrhenius type representation of the exfoliation time at varying temperatures. (E) and (F) TEM images of exfoliated TaO_3_ nanosheets on a carbon TEM grid.

Firstly, the different exfoliation agents and molar ratios were examined. TBAOH is the most commonly used exfoliation agent with bulky butyl groups surrounding a central ammonium ion. However, a recent paper reported that TBPOH, where the nitrogen atom is replaced by a phosphorus atom, could induce exfoliation more easily.^22^ The TBP^+^ ion is relatively comparable with TBA^+^, but the larger size of P and its smaller electronegativity may help to destabilize and delaminate the layered structure more readily. Since protonated tantalum oxide takes a long time to exfoliate, the use TBPOH could be one way to reduce the exfoliation time and yield a higher concentration of nanosheets, which would also be interesting for various deposition techniques, including inkjet printing. Since the surface charge density of TaO_3_^−^ sheets is relatively high (−1.6 C m^−2^), HTaO_3_ is a difficult compound to exfoliate, and it is desirable to explore different exfoliation agents at different molar ratios to facilitate the exfoliation process. It has been reported that optimal molar ratios between exfoliation agent and intergallery protons is between 0.5 and 5.^23^ In order to compare the commonly used TBAOH and the lesser-known TBPOH, we employed molar ratios of 1 : 1 and 2 : 1 (exfoliation agent: protons). Fig. 2(A) shows the LUPs as a measure of nanosheet concentrations using both exfoliation agents at these different ratios. TBPOH showed the largest LUP at a 2 : 1 ratio. This was also the only nanosheet dispersion with which a fully surface-covering monolayer was formed *via* LB deposition on a silicon substrate; LB experiments with TBAOH did not yield significant concentrations of nanosheets on the substrates, and neither did the solution with TBPOH at a ratio of 1 : 1. We therefore selected the exfoliation agent TBPOH at a molar ratio of 2 : 1 to HTaO_3_ for all further experiments.

The next step in the optimization process was to reduce the exfoliation time by increasing the temperature. Exfoliation reactions were conducted at temperatures between room temperature and 80 °C. Fig. 2(B) shows the normalized lift-up points for the tantalum oxide nanosheets *versus* reaction time at 3 different temperatures.

A peak concentration was observed after a given period of time at all three temperatures, in close agreement with earlier findings on Ti_1−*x*_O_2_ nanosheets.^13^ Initially, the interfacial concentration of nanosheets increased with time as a result of ongoing exfoliation. After reaching a maximum, the concentration of nanosheets decreased with time. The mechanism for exfoliation and restacking, proposed by Yuan *et al.*,^13^ seems to be confirmed in these experiments: firstly, the layered HTaO_3_ crystals are exfoliated by the acid–base reaction between interlayer protons and hydroxyl ions from TBPOH. The rate of this reaction is proportional to the HTaO_3_ concentration in the suspension, *i.e.* a first order reaction in [HTaO_3_]. However, two unilamellar nanosheets are able to restack, *i.e.* following a second order reaction rate. The exfoliation step therefore dominates the process at low nanosheet concentration, whereas the restacking step only becomes significant later when the measured concentration of (single) nanosheets is higher. The nanosheet concentration maximum occurs when these two reactions have the same rate, after which the restacking reaction starts to dominate and the nanosheet concentration at the interface declines.

Less time was needed to reach the highest LUPs at higher temperatures; reaching the maximum took 3 weeks at room temperature, while at 80 °C it only took 36 hours (see Fig. 2(C)). This observation shows that by increasing the reaction temperature, the reaction time reduced significantly.

Employing an exfoliation reaction temperature of 80 °C for 36 h, and subsequently cooling the nanosheet solution to room temperature allowed us to postpone and flatten the gradual decrease in the concentration of nanosheets at the liquid–air interface. This allowed us to generate larger volumes of stock solution, with a constant concentration of nanosheets lasting for more than a week, enough to carry out multiple depositions (Fig. S2, ESI†). TEM images confirmed the successful exfoliation of the layered HTaO_3_ parent compound into unilamellar sheets (Fig. 2(E) and (F)). Various sheet sizes can be observed laying next to or on top of each other turbostratically.

Arrhenius-type behavior was assumed to calculate the apparent overall activation energy of the exfoliation and restacking process. The Arrhenius equation assumes that 
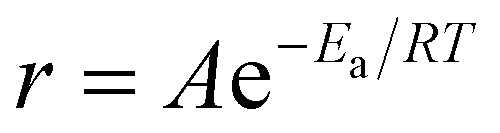
, where *r* is the rate, *A* is a pre-exponential factor, *E*_a_ is the apparent activation energy, *R* is the gas constant and *T* is the temperature in Kelvin.

We defined the rate as the ratio of concentration over time needed to arrive at the surface concentration maximum. Fig. 2(D) shows an Arrhenius-type representation of the natural logarithm of the reaction rate *versus* 1/*T*. The slope of the curve corresponds with an apparent activation energy of 39.7 kJ mol^−1^ ± 25%. This value is of the same order of magnitude as the activation energy needed to obtain graphene from graphite using constant current or constant voltage: 20.6–23.1 kJ mol^−1^.^24^ Yet, its quantitative value is significantly higher, and it is also considerably higher than the activation energy of 27.3 kJ mol^−1^ for the exfoliation of RuO_2_.^16^ The sequence in apparent activation energies of exfoliation, *i.e.* graphene < RuO_2_ nanosheets < TaO_3_ nanosheets is in qualitative good agreement with the differences in the cohesive binding energies between graphene nanosheets, which are dominated by van der Waals forces, and the stronger electrostatic interactions between H^+^/RuO_2_^−^ and H^+^/TaO_3_^−^, respectively. The exfoliation time needed to form RuO_2_ nanosheets at room temperature under standard conditions is roughly two weeks,^25^ which is somewhat shorter than reported previously for TaO_3_ nanosheets.^14^ Shorter exfoliation times under otherwise similar conditions indicate an easier to exfoliate material, which was confirmed by the experimental activation energies for exfoliation. Moreover, taking into consideration that the surface charge density of TaO_3_^−^ nanosheets (−1.6 C m^−2^) is considerably higher than that of RuO_2_^−^ nanosheets (−0.46 C m^−2^), the difference in apparent activation energies (39.7 kJ mol^−1^*versus* 27.3 kJ mol^−1^ respectively) confirms our hypothesis that layered materials with higher surface charge densities are harder to exfoliate.

### LB deposition of TaO_3_ nanosheets

**Fig. 3:**
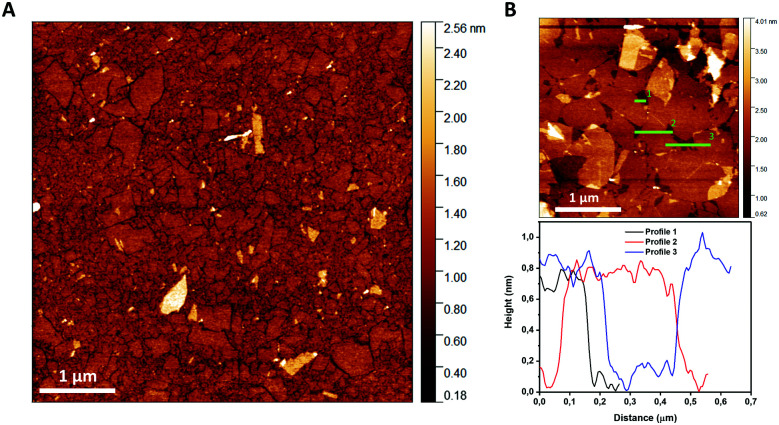
(A) AFM image of TaO_3_ nanosheets deposited on a silicon substrate using LB deposition. (B) Different AFM image including three height profiles showing a nanosheet thickness of ∼0.75nm.

Using TBPOH to HTaO_3_ at a molar ratio of 2 : 1 and an exfoliation temperature of 80 °C, dispersions were made with which densely covered LB films of TaO_3_ nanosheets on silicon substrates could be realized. These substrates are ultra-flat and allow precise determination of coverage and thickness by AFM analysis. The surface coverage by TaO_3_ nanosheets and the thickness of these nanosheets on the silicon substrate are shown in Fig. 3(A) and (B), respectively.

Fig. 3(A) shows a substrate coverage of >95% after deposition of a single layer LB-derived nanosheet film and its subsequent heat treatment at 350 °C in air, to remove all organic residues. The experiment clearly shows that the complete surface (>95%) can be covered by LB deposition. The lateral dimensions of TaO_3_ nanosheets are about 100 × 100 nm, while the largest nanosheets are about 500 nm in diameter. The nanosheet thickness is estimated to be ∼0.75 nm. This is slightly thinner than as reported in literature (∼1 nm).^7^ However, when nanosheets are deposited on a substrate using the LB process, there will always be a layer of molecules between the nanosheets and the substrate, for example water and TBPOH moieties. The fact that a heat treatment was employed in our experiments between the LB process and the AFM analysis to remove such molecules, or at least most of them, might explain the smaller thickness.

### Inkjet printing of TaO_3_ nanosheets

**Fig. 4:**
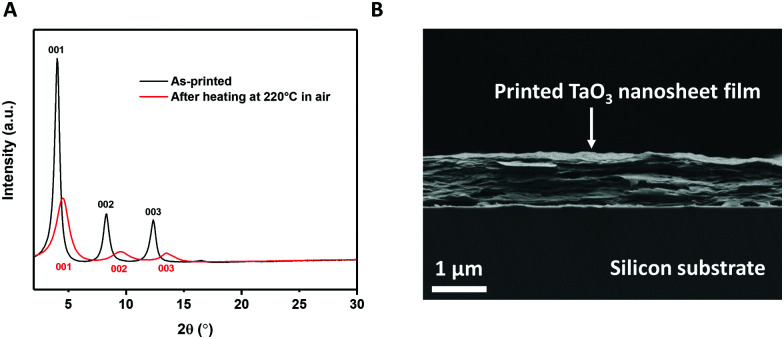
(A) X-Ray diffractogram of printed TaO_3_ nanosheets before and after heat treatment. (B) Cross-sectional SEM image of printed TaO_3_ nanosheets on silicon.

The same procedure of making TaO_3_ nanosheets in solution for LB deposition was also used to make inks for inkjet printing. These inks need to have sufficiently high concentrations of nanosheets to achieve full coverage of the printed pattern in a single pass.^26^ We inkjet printed TaO_3_ nanosheets on ultra-flat silicon substrates to allow characterization using XRD and SEM. The X-ray diffractograms of TaO_3_ nanosheet films on silicon, before and after a 1 h heat treatment at 220 °C in air are shown in Fig. 4(A). The heat treatment temperature is lower than as applied to the LB-films described above. In general, lower annealing temperatures are desirable because they allow a wider range of substrate choices. The corresponding SEM image of the heat treated sample is shown in Fig. 4(B). Various thicknesses of TaO_3_ thin films were printed, in Fig. 4(B) the thickness is ∼900 nm.

The diffractograms provide a clear confirmation of a turbostratic layered system, with unilamellar nanosheets laying on top of each other with their basal planes out of alignment. Since the nanosheets are not oriented in-plane with respect to each other, only the (00x) diffraction peaks are present (see Fig. 4(A)). None of the reflections of unexfoliated material, present in Fig. 1(B), were present in Fig. 4(A), which suggests that only unilamellar nanosheets were present in the printing ink. The (001), (002) and (003) reflections were clearly present both before and after heat treatment. However, after heat treatment, the same three reflections were found at higher 2*θ* and were slightly broader. This 2*θ* increase indicates a shrinkage of interlayer distance by 2.4 Å, from 21.8 Å to 19.4 Å. This value of 2.4 Å is comparable to roughly one monolayer of intercalated molecules, such as water or TBP^+^. The broader peaks indicate a more heterogeneous structure in terms of interlayer spacings. The FWHM of the as-printed sample is 0.25°, compared with 0.8° for the heat treated sample. The reduction of interlayer distance and the broadening of the peaks can both be explained by the effects of heat treatment. During the heat treatment the residual solvent molecules trapped between nanosheets evaporated, causing the nanosheets to come closer to each other. The evaporating solvent was transported out of the structure, thereby possibly causing the stacks of nanosheets to become slightly more disordered in that process. This assumption was confirmed in the cross sectional SEM image in Fig. 4(B) where a slight wavy pattern can be seen.

### Electrical bandgap measurements of single TaO_3_ nanosheets

**Fig. 5:**
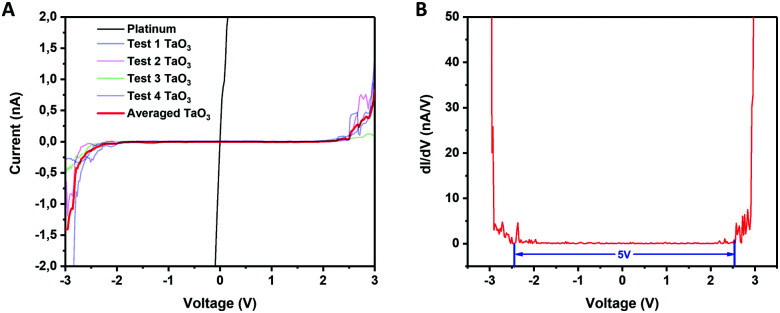
(A) Current *versus* voltage for measurements of platinum (substrate) and nanosheets. (B) d*I*/d*V versus* voltage (*V*) of the same measurements.

LB films of TaO_3_ nanosheets deposited on platinum-coated silicon substrates were used to electrically measure the bandgap of individual nanosheets by scanning tunneling microscopy (STM). The substrate was partially covered with TaO_3_ nanosheets, and STM measurements were done on and off single nanosheets. Fig. 5(A) shows the current *versus* voltage measurements (averaged over 4 measurements) and Fig. 5(B) shows the d*I*/d*V versus* voltage curve, which is a representation of the density of states in 2D TaO_3_. The *I–V* curve of the STM tip in direct contact with the platinum-coated silicon substrate is shown as a reference.

The electrically measured bandgap of a single TaO_3_ nanosheet is ∼5 eV, which is in accordance with optically measured bandgaps for tantalum oxide nanosheets in suspension.^14^ The three-dimensional Ta_2_O_5_ phase has an average bandgap of 4 eV,^8^ which is considerably lower than the bandgap found for these 2-dimensional TaO_3_ nanosheets. Moreover, the bandgap for RbTaO_3_ has been reported to be 3.5^9,10^ and 4.3 eV,^11^ which values are also notably lower than the bandgap found for the 2D counterparts in this study. Possibly, when going from a 3D to a 2D structure, the quantum confinement of electrons in one dimension may have led to an increase of the bandgap. The same phenomenon has also been reported other layered materials, notably layered titanates with a similar nanosheet thickness well below 1 nm just like TaO_3_ has.^27,28^ Our study thereby shows that by exfoliating 3D layered TaO_3_ based materials into their 2D counterparts may increase their bandgap significantly, making these materials more electrically insulating. This bandgap increase is interesting for applications in, for example, solid-state electrolytes.

## Conclusions

TaO_3_ nanosheets were formed by complete exfoliation of HTaO_3_ at 80 °C in just 36 h as opposed to three weeks at room temperature. TBPOH was used as exfoliating agent in a molar ratio TBPOH: HTaO_3_ of 2 : 1. Using the same approach for other layered materials that are difficult or currently impossible to exfoliate, could open up the way for new 2D materials with new interesting properties. Both monolayer films of nanosheets and inkjet printed multilayer films were realized. TaO_3_ nanosheets are inkjet printed on silicon substrates and X-ray diffractograms suggest a highly ordered layered system. The bandgap of a single TaO_3_ nanosheet was electrically measured using STM and was found to correspond to 5 eV. This indicates that the TaO_3_ nanosheets are insulating, which is one of the requirements of (solid) electrolytes in batteries.

## Conflicts of interest

There are no conflicts to declare.

## Supplementary Material

TC-009-D1TC00801C-s001
