# Supplementary material for: Long-range ordering of two-dimensional wide bandgap tantalum oxide nanosheets in printed films
Source: J Mater Chem C Mater. 2021 Apr 9;9(17):5699–705. doi: 10.1039/d1tc00801c (PMC8101408; doi:10.1039/d1tc00801c)
Supplement: TC-009-D1TC00801C-s001 [file TC-009-D1TC00801C-s001.pdf]

Electronic Supplementary Material (ESI) for Journal of Materials Chemistry C.
This journal is © The Royal Society of Chemistry 2021

Electronic Supplementary Information

Long-range ordering of two-dimensional wide bandgap tantalum oxide nanosheets in printed films

Melvin A. Timmerman,[a] Rui Xia,[a] Yang Wang,[a] Kai Sotthewes,[a] Mark Huijben,*[a] and Johan E. ten Elshof *[a]

[a] University of Twente, MESA+ Institute for Nanotechnology, P.O. Box 217, 7500 AE, Enschede, the Netherlands

Authors for correspondence: j.e.tenelshof@utwente.nl, m.huijben@utwente.nl

1. Determination of Rb content after protonation

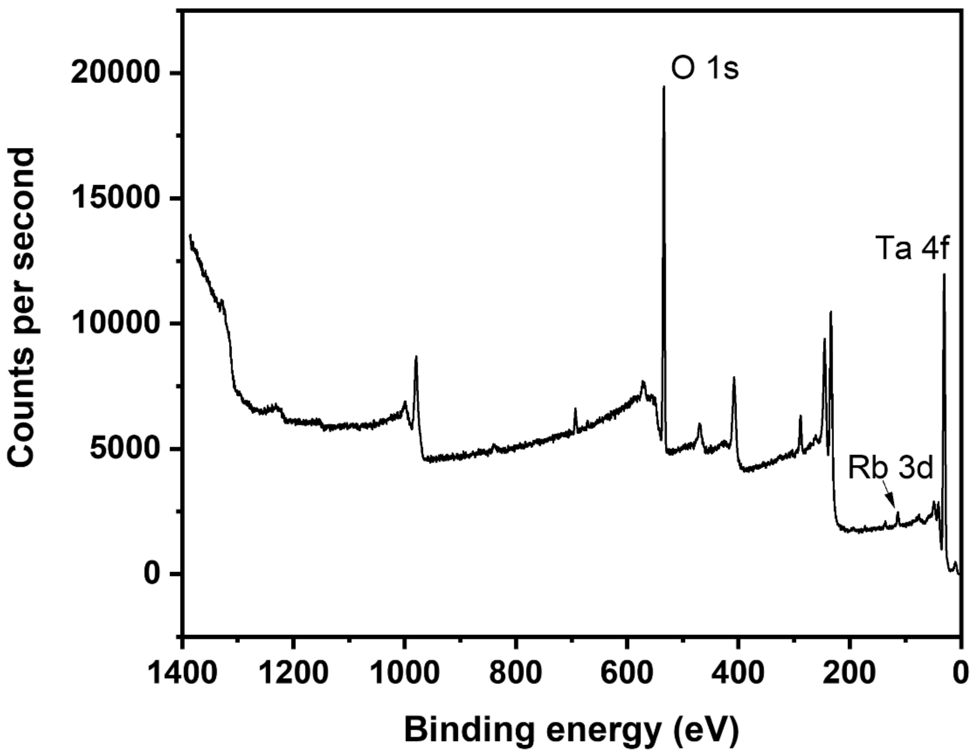

Figure S1 XPS data of an inkjet printed TaO_3 film on silicon.

The XPS spectrum in Figure S1 shows the presence of Rb atoms in the structure. To quantify the amount of Rb in the structure, XRF was used to measure the bulk composition as opposed to the surface sensitive XPS measurement.

The weight and atomic concentration of Ta and Rb are shown in Table S1 below.

Table S1 calculated concentrations of Ta and Rb from the XRF spectrum.

Formula	Weight %	Atom %	Stat. error (%)
Ta	97.90	95.7	1.73
Rb	2.06	4.3	10.90

This results in an atomic ratio of Ta to Rb of 22.3 : 1 which shows that 95.6% of the Rb atoms have been removed/replaced upon protonation.

2. Determination of the stability of the interface concentration of TaO_3 nanosheets at room temperature

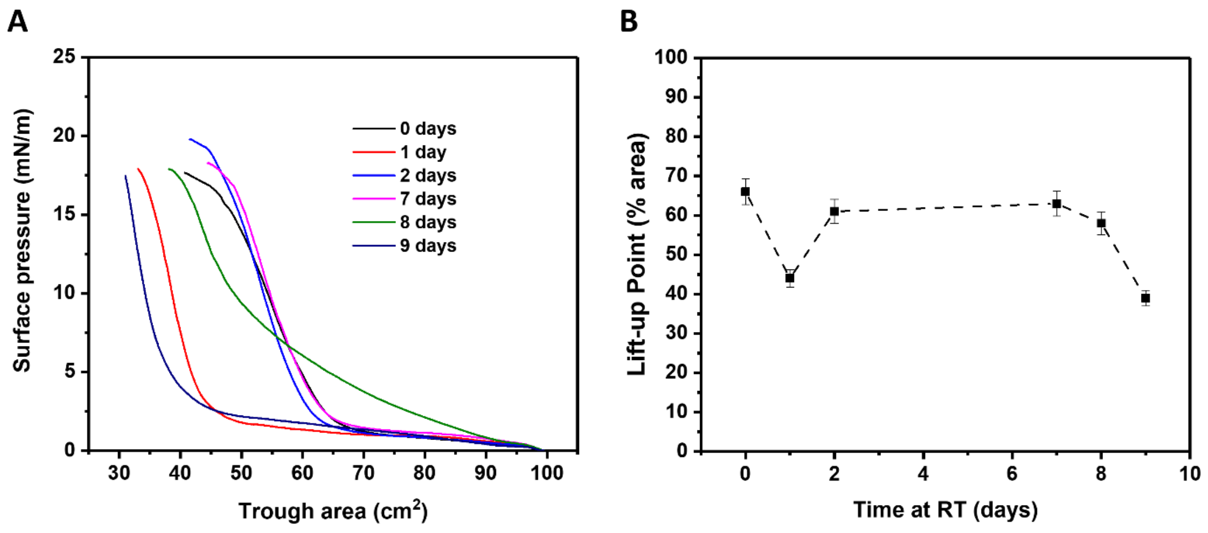

Figure S2 (a) Surface pressure versus trough area during Langmuir-Blodgett depositions of various times at room temperature after exfoliation. (b) Lift-up point versus time at room temperature for the different Langmuir-Blodgett depositions.

In Figure S2 (a) the surface pressure isotherms of various Langmuir-Blodgett depositions are plotted versus the surface area of the trough. The corresponding lift-up points of the depositions are plotted versus ageing time at room temperature in Figure S2 (b). The exfoliation reaction was employed at 80°C, after which the solution was kept at room temperature for several days. The dashed line serves as a guide to the eye. The lift-up points of the various depositions stayed stable until ~9 days ageing at room temperature after which it declined. The lift-up points, which scale with the nanosheet solution at the air-water interface, can thus be maintained stable at room temperature for more than a week.
